# Intrahepatic Lymphoid Follicles Comprising T and B Cells Mimic Hepatocellular Carcinoma in a Hepatitis B Patient

**DOI:** 10.3390/ijms26104823

**Published:** 2025-05-18

**Authors:** Ji Yeon Lee, Jaejun Lee, Pil Soo Sung

**Affiliations:** 1 Department of Internal Medicine, Seoul St. Mary’s Hospital, College of Medicine, The Catholic University of Korea, Seoul 06591, Republic of Korea; jsaenaen@naver.com; 2 Division of Gastroenterology and Hepatology, Department of Internal Medicine, Seoul St. Mary’s Hospital, College of Medicine, The Catholic University of Korea, Seoul 06591, Republic of Korea; pwln0516@gmail.com

**Keywords:** isolated lymphoid follicles, hepatocellular carcinoma, biopsy, atezolizumab

## Abstract

Isolated intrahepatic lymphoid follicles (ILFs), also referred to as reactive lymphoid hyperplasia, are rare benign lymphoid proliferations that can closely mimic hepatocellular carcinoma (HCC) on imaging. This case highlights the diagnostic complexity of hepatic mass lesions in chronic hepatitis B patients, particularly when radiologic and serologic features raise concern for malignancy. A 60-year-old man with chronic hepatitis B presented with a liver mass, elevated alpha-fetoprotein levels, and imaging findings of heterogeneous arterial enhancement, all suggestive of HCC. Despite initial treatment with atezolizumab plus bevacizumab, the lesion progressed, leading to an extended left hepatectomy. Histopathological examination revealed well-formed lymphoid follicles with reactive germinal centers, without evidence of malignancy or granulomatous inflammation. Serum IgG was elevated, and ANA was positive, supporting the possibility of an underlying immune-mediated process. The patient showed clinical and serologic improvement following corticosteroid therapy, with no evidence of recurrence at 10-month follow-up. This case underscores the importance of histopathological confirmation in hepatic masses with atypical features and highlights the need to consider benign immune-related mimickers in the differential diagnosis, particularly in the era of immunotherapy.

## 1. Introduction

Isolated lymphoid follicles (ILFs) or reactive lymphoid hyperplasia (RLH) of the liver is a rare, benign, and often incidental histopathological finding characterized by the proliferation of non-neoplastic, polyclonal lymphoid cells forming follicles with reactive germinal centers [1,2]. Although ILFs in the liver are frequently observed in patients with chronic hepatitis C, they have also been identified in various autoimmune and inflammatory liver diseases, including autoimmune hepatitis and primary biliary cholangitis, suggesting an immunopathological basis for their development [3]. The exact etiology remains unclear, but chronic antigenic stimulation, immune dysregulation, or autoimmune activation are hypothesized as potential contributors [4,5].

Imaging characteristics of hepatic ILFs are often non-specific and can mimic malignant hepatic neoplasms such as hepatocellular carcinoma (HCC) or lymphoma. On MRI, ILF lesions may display hyperintensity on T2-weighted images, diffusion restriction on DWI, and perinodular enhancement in delayed phases, findings that can overlap with early-stage HCC [6,7]. Furthermore, increased uptake on FDG-PET may further confound the diagnosis [8]. In the absence of established cirrhosis or elevated tumor markers, such lesions can pose a significant diagnostic challenge, often leading to surgical intervention for definitive diagnosis.

In this report, we present a diagnostically challenging case of ILFs in a patient with chronic hepatitis B, who presented with a large hepatic mass and elevated tumor markers suggestive of HCC. Radiologic progression during systemic therapy raised concerns for malignancy, and histopathological evaluation demonstrated RLH with no malignant features. This case underscores the critical role of histopathological assessment in distinguishing benign lymphoid proliferations from malignant hepatic tumors and contributes novel insights into the immunopathological spectrum of hepatic pseudotumors.

## 2. Case Presentation

A 60-year-old man with a history of diabetes mellitus underwent medical check-up at a primary care clinic, where he was found to have esophageal varices and an elevated alpha-fetoprotein (AFP) level. The patient subsequently presented with a fever of 38.7 °C but reported no abdominal pain or weight loss. His physical examination was unremarkable. Given these findings, he was referred to the hepatology division at a tertiary hospital for further evaluation. Initial laboratory tests were positive for hepatitis B surface antigens but negative for hepatitis B e antigens. Hepatitis B virus DNA titers were 2172 IU/mL. Liver enzymes, including aspartate aminotransferase and alanine aminotransferase, as well as prothrombin time and complete blood count, were within normal limits, while alkaline phosphatase (122 IU/L; normal: 35–104 IU/L) and gamma-glutamyl transpeptidase (155 U/L; normal: 9–85 U/L) showed mild elevations. Serum tumor marker analysis revealed elevated levels of AFP at 13.2 ng/mL (normal: <8.1 ng/mL), carbohydrate antigen 19-9 at 35 U/mL (normal: <34 U/mL), and carcinoembryonic antigen at 5.11 ng/mL (normal: <3.8 ng/mL). Anti-nuclear antibodies were positive at a titer of 1:160, and immunoglobulin G (IgG) was markedly elevated at 3006 mg/dL (normal range: 700–1600 mg/dL). Other autoimmune markers such as anti-neutrophil cytoplasmic Ab, anti-mitochondrial Ab, anti-smooth muscle Ab, anti-double stranded DNA Ab, anti-SSA Ab, and anti-liver kidney microsome type 1 Ab were all negative.

**Figure 1:**
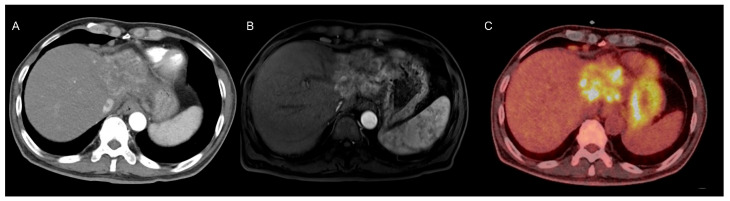
Multimodal Imaging of the Tumorous Lesion. (**A**) Computed tomography showing heterogeneous arterial enhancement within the lesion. (**B**) Gadoxetic-acid-enhanced liver magnetic resonance imaging of arterial phase demonstrating heterogeneous enhancement of the lesion. (**C**) Positron emission tomography–computed tomography of the liver demonstrating intense fluorodeoxyglucose uptake within the lesion.

A liver dynamic CT scan revealed a 6.2 cm × 6.1 cm × 4.8 cm heterogeneously enhancing mass in the left lateral section of the liver, along with multiple enlarged lymph nodes in the right pericardiophrenic and porta hepatis areas (Figure 1A). Gadoxetic-acid-enhanced liver MRI showed a 7.8 cm hyperenhancing mass in the left lateral section during the arterial phase of dynamic imaging (Figure 1B). Based on the radiological findings and elevated tumor marker levels, the patient was diagnosed with HCC, modified Union for International Cancer Control (mUICC) stage IVA.

Treatment with atezolizumab bevacizumab was initiated, along with antiviral treatment using tenofovir alafenamide to ensure virologic suppression of the hepatitis B virus. However, follow-up liver MRI after two cycles revealed tumor progression, with the lesion increasing from 7.8 cm to 8.8 cm in its longest axis. A multidisciplinary consultation was conducted, and surgical resection was recommended as the next treatment modality given the progression despite atezolizumab bevacizumab therapy. Preoperative positron emission tomography–computed tomography revealed intense heterogeneous fluorodeoxyglucose uptake within the tumor (Figure 1C). The patient subsequently underwent extended left hepatectomy.

Histological examination of the adjacent non-tumor liver tissue revealed architectural distortion and fibrous septa consistent with advanced fibrosis. Mild periportal inflammation was noted, but there were no features strongly indicative of autoimmune hepatitis.

**Figure 2:**
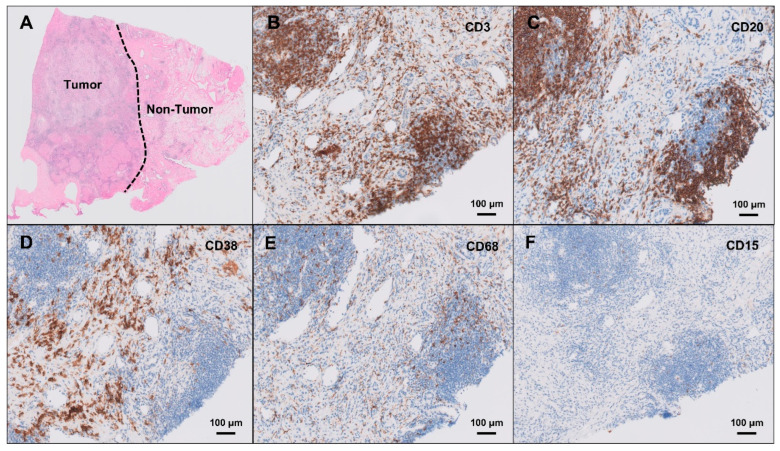
Representative histological image of the liver demonstrating granulomatous inflammation. (**A**) Hematoxylin- and eosin-stained histological image. (**B**) CD3 immunohistochemical staining highlighting T lymphocytes (scale bar: 100 μm, 200× magnification). (**C**) CD20 immunohistochemical staining highlighting B lymphocytes (scale bar: 100 μm, 200× magnification). (**D**) CD38 immunohistochemical staining highlighting plasma cells (scale bar: 100 μm, 200× magnification). (**E**) CD68 immunohistochemical staining highlighting macrophages (scale bar: 100 μm, 200× magnification). (**F**) CD15 immunohistochemical staining highlighting neutrophils (scale bar: 100 μm, 200× magnification).

Histopathological examination of the resected lesion revealed well-formed intrahepatic lymphoid follicles with reactive germinal centers, accompanied by dense lymphoplasmacytic infiltration and a prominent ductular reaction, without evidence of granulomatous inflammation or malignancy. The hematoxylin- and eosin-stained histological image is presented in Figure 2A. Immunohistochemical staining demonstrated that these lymphoid follicles were primarily composed of CD3-positive T cells (Figure 2B) and CD20-positive B cells (Figure 2C), indicating structured T- and B-cell zonation within the lymphoid aggregates. CD38-positive plasma cells (Figure 2D) were predominantly distributed in the peri-follicular hepatic parenchyma, rather than within the lymphoid follicles themselves, suggesting localized humoral immune activation. CD68-positive macrophages (Figure 2E) were relatively sparse within the lymphoid follicles, and CD15-positive neutrophils (Figure 2F) were minimally present. IgG4 was positive in some of the IgG-positive plasma cells, and anaplastic lymphoma kinase (ALK) staining was negative, effectively ruling out an ALK-positive inflammatory myofibroblastic tumor, ALK-positive histiocytosis, and ALK-positive lymphoma. No tumor cells were identified, and extensive work-up for infectious causes, including tuberculosis, fungal infections, and other atypical microorganisms, was negative. Taken together, the histological and immunohistochemical findings were consistent with ILFs, a rare form of reactive lymphoid hyperplasia mimicking hepatic neoplasia.

Following surgical resection and histopathological confirmation of ILFs, the patient was treated empirically with antibiotics and corticosteroids for three weeks. The steroid regimen started with methylprednisolone 0.5 mg/kg and was tapered down to prednisolone (5 mg). The IgG levels declined significantly from 3006 mg/dL to 777 mg/dL after surgical resection, and the ANA titer decreased from 1:160 to 1:80. Follow-up dynamic liver CT performed 10 months after surgery showed no evidence of lesion recurrence. Given the elevated IgG levels and partial IgG4 positivity among plasma cells, a low dose of prednisolone (2.5 mg) was maintained in consideration of a possible immune-mediated etiology.

## 3. Discussion

This case emphasizes several critical considerations in evaluating hepatic masses. The initial diagnosis of HCC was strongly supported by the patient’s clinical background of chronic hepatitis B, elevated tumor markers, and imaging findings of the liver mass. However, lack of response to chemotherapy and the subsequent histopathological diagnosis of benign ILFs underscore the essential role of tissue diagnosis, particularly in atypical or treatment-refractory hepatic lesions.

ILFs, also referred to as RLH, are characterized by non-clonal, polytypic lymphoid cell proliferation with organized germinal centers. Although the liver is not typically considered a site of lymphoid tissue development, it can exhibit ectopic lymphoid structures under conditions of chronic immune stimulation [5]. In fact, RLH of the liver has been reported in association with chronic liver diseases, including primary biliary cirrhosis, viral hepatitis, and nonalcoholic steatohepatitis [2]. Lymphoid follicles are generally found in livers with chronic disease, but not in histologically normal livers, further supporting the relationship between chronic hepatic inflammation and the development of ILFs [3,4,5,9].

Differentiating ILFs from hepatic malignancy remains a significant challenge, as ILFs may exhibit imaging characteristics that closely resemble primary liver cancer. Lesions may appear hypointense on T1-weighted imaging and hyperintense on T2-weighted sequences, with arterial phase hyperenhancement and portal venous washout, a radiologic hallmark commonly associated with HCC [1,10]. This overlap, especially in the absence of chronic liver decompensation or markedly abnormal tumor markers, can obscure the correct diagnosis. Additionally, the well-demarcated mass-like presentation seen in this patient is atypical for many autoimmune or inflammatory liver diseases, including IgG4-related hepatic disease, which more frequently manifests as diffuse or periductal infiltrates [11]. Given the presence of portal hypertension and a history of chronic hepatitis B in our case, radiologists may have been predisposed to interpret the lesion as HCC. Such diagnostic bias highlights the importance of considering benign mimickers like ILFs in patients with underlying liver disease.

From an immunopathological perspective, ILFs represent localized, polyclonal proliferations of lymphoid tissue composed of well-formed germinal centers and structured B- and T-cell zones. While their precise etiology remains unclear, ILFs are hypothesized to arise from chronic antigenic stimulation, autoimmune activity, or localized immune dysregulation [4,5]. In our case, the presence of CD3-positive T cells and CD20-positive B cells forming follicular architecture, alongside IgG4-positive plasma cells and an elevated serum IgG level, point toward an underlying immune-mediated mechanism rather than an infectious or neoplastic process. The observed reduction in serum IgG and ANA titers following corticosteroid therapy further supports this interpretation. Although ILFs are generally benign, their presentation as mass-forming lesions can prompt invasive interventions and misdirected therapies if not accurately diagnosed. Histological and immunophenotypic confirmation, including assessment of clonality and the exclusion of lymphoma or malignancy, is therefore crucial.

Management of hepatic ILFs is not standardized due to their rarity and often incidental discovery. Although no standardized treatment exists for hepatic ILFs, corticosteroids may be considered in select cases with serologic or histologic features suggestive of an immune-mediated process. The normalization of serum IgG levels and absence of recurrence on follow-up imaging support a self-limited, immune-reactive process rather than persistent or progressive pathology.

## 4. Conclusions

This case highlights the importance of considering ILFs as a potential cause of hepatic mass lesions, particularly when clinical, serologic, or imaging features are not typical for malignancy. Given the significant overlap in radiologic appearance between ILFs and hepatocellular carcinoma, histopathological evaluation remains essential, especially in cases showing progression despite standard treatment. As immune-based therapies become more widely used in hepatology and oncology, the potential for immune-mediated hepatic lesions to mimic malignancy further emphasizes the need for tissue diagnosis. This case underscores the value of a thorough, multidisciplinary assessment in the diagnostic workup of atypical liver lesions.

## Data Availability

The original contributions presented in the study are included in the article. Further inquiries can be directed at the corresponding author.

## Abbreviations

AFP, alpha-fetoprotein; CT, computed tomography; HCC, hepatocellular carcinoma; ILF, isolated lymphoid follicle; MRI, magnetic resonance imaging; mUICC, modified Union for International Cancer Control; RLH, reactive lymphoid hyperplasia.
